# The development and optimisation of a primary care-based whole system complex intervention (CARE Plus) for patients with multimorbidity living in areas of high socioeconomic deprivation

**DOI:** 10.1177/1742395316644304

**Published:** 2016-04-10

**Authors:** Stewart William Mercer, Rosaleen O'Brien, Bridie Fitzpatrick, Maria Higgins, Bruce Guthrie, Graham Watt, Sally Wyke

**Affiliations:** General Practice and Primary Care, University of Glasgow, United Kingdom of Great Britain and Northern Ireland

**Keywords:** Primary care, multimorbidity, deprivation, complex intervention, quality of life

## Abstract

**Objectives:**

To develop and optimise a primary care-based complex intervention (CARE Plus) to enhance the quality of life of patients with multimorbidity in the deprived areas.

**Methods:**

Six co-design discussion groups involving 32 participants were held separately with multimorbid patients from the deprived areas, voluntary organisations, general practitioners and practice nurses working in the deprived areas. This was followed by piloting in two practices and further optimisation based on interviews with 11 general practitioners, 2 practice nurses and 6 participating multimorbid patients.

**Results:**

Participants endorsed the need for longer consultations, relational continuity and a holistic approach. All felt that training and support of the health care staff was important. Most participants welcomed the idea of additional self-management support, though some practitioners were dubious about whether patients would use it. The pilot study led to changes including a revised care plan, the inclusion of mindfulness-based stress reduction techniques in the support of practitioners and patients, and the stream-lining of the written self-management support material for patients.

**Discussion:**

We have co-designed and optimised an augmented primary care intervention involving a whole-system approach to enhance quality of life in multimorbid patients living in the deprived areas. CARE Plus will next be tested in a phase 2 cluster randomised controlled trial.

## Introduction

Multimorbidity is usually defined as the coexistence of two or more long-term conditions within an individual, and is increasing common in populations across the world.^1^ In a large nationally representative study of the epidemiology of multimorbidity in Scotland, we found that multimorbidity was present in almost 25% of the population.^2^ In the 10% most deprived areas, multimorbidity occurred 10–15 years earlier than in the 10% least deprived areas.^2^ A similar social gradient has also been found in several other studies.^1,3^ The burden of multimorbidity is higher in those living in more deprived areas in terms of effect on quality of life.^4^ The combination of mental and physical conditions (mental–physical multimorbidity) is 2–3 fold higher in the most deprived compared with the least deprived areas^2^ and this is most pronounced in younger patients.^5^ Mental–physical multimorbidity is associated with high levels of unplanned hospital admissions in the deprived areas.^6^ Primary care staff recognise the ‘endless struggle’ that multimorbid patients living in the deprived areas face in terms of managing daily life.^7^ Patients have described in detail the burdens that managing the ‘everyday life work’ and living with complex social, psychological and physical problems can create.^8^

The evidence-base for how best to manage patients with multimorbidity in primary care is very limited, especially in the context of deprivation.^9^ In view of this, we established the ‘Living Well with Multimorbidity’ research programme in Scotland to develop a primary care-based complex intervention for patients with multimorbidity in areas of high deprivation, using the MRC guidance on developing complex interventions.^10^ We defined the scale of the problem and the target population^2^ and carried out qualitative research with primary care practitioners working in the deprived areas and with multimorbid patients living in such areas^7,8^ in order to explore the challenges of ‘living well’ and how primary care might better respond. From this baseline work, plus consideration of the wider literature on patient-centred care and self-management support, and input and discussion with an international advisory panel consisting of experts in the field of complex intervention design, we identified the possible components of a whole systems approach. These were:

- System changes to allow longer consultations and relational continuity (seeing the same practitioner each time)
- Practitioner training and support to deliver structured care in those longer consultations including care-planning and
- Additional self-management support for patients.

This paper describes the further development and co-design of the intervention based on qualitative focus group discussions with patients, patient representatives and primary care providers working in areas of very high deprivation, and its optimisation following piloting in two practices located in the high deprivation areas.

## Methods

Separate approvals were obtained from the NHS Local Research Ethics Committee and NHS Research & Development for each phase of the study. Written informed consent was obtained from all the study participants before data collection commenced.

## Defining and developing the intervention

Possible components of the proposed intervention were identified from the literature and by expert consensus, and provided material for six group discussions with 32 participants in all. These took the form of interactive workshops in which participants were first given a summary of what was already known by SWM, and then took part in a group discussion which provided the data reported in this paper. Two of the workshops were with patients; one with 5 participants and the other with 3 participants, each from one of two different practices in the deprived areas of Glasgow. Four of the patients had taken part in previous interviews^8^ and had consented to future interviews, and four were recruited by the practices based on eligibility criteria (two or more long-term conditions, and aged 30–65 years). The ages of those who took part ranged from 42 to 65 years (mean 54 years), and six out of the eight were female. All had multimorbidity including conditions such as stroke, heart disease, chronic back pain, arthritis, depression, asthma, cancer and hypertension.

Two of the workshops were with general practitioners (GPs) working in the deprived areas; one with 3 participants and the other with 9 participants, all representing different practices. One workshop was with practice nurses (PNs) working in the deprived areas; 4 participants representing different practices. The remaining workshop was with members of different third sector organisations; 8 participants representing charitable organisations concerned with a range of long-term conditions brought together by the Alliance for Health and Social Care (formerly the Long-Term Conditions Alliance Scotland).

In each workshop, SWM presented the background to the study and the evidence to date, including the epidemiological^2^ and qualitative ‘baseline’ studies^7,8^ completed in the first phase of the Living Well with Multimorbidity Programme. He then outlined the proposed intervention and its components. This information was conveyed by means of a power-point talk lasting 15–20 min at the start of each group together with printed hand-outs of the slides. In the GP and PN focus groups, he also presented two models that could be used in the longer consultations to help structure them; the 5 A’s approach,^11^ which is a tool to support practitioners in delivering self-management support, and the CARE Approach^12,13^ which is a holistic approach to empathic, patient-centred care.

One qualitative researcher attended the meetings in addition to SWM and observed responses to the presentations, took field notes and led the subsequent group discussions; RO attended one meeting, and MH attended the others. The discussions were audio recorded with permission and transcribed verbatim. The transcripts were subsequently coded and analysed by BF using the framework approach.^14^ Coding focused on participants’ views on each component of the whole-system intervention: time and relational continuity, practitioner training and support and self-management support. A sub-sample of the transcripts was double coded and discrepancies were settled following discussion between the two coders (BF and SWM). Findings were then discussed by the research team and formed the basis of the second iteration of the intervention.

## Piloting and optimising the intervention

The second iteration of the proposed intervention was then piloted in two high deprivation practices over a 3-month period. Practice A had 3500 patients registered, with 7 GP Partners (most working part-time) and 2 PNs. Practice B had 4000 patients, 4 GP partners and 1 PN. In Practice A, all the GP Partners and PNs participated in the study and in Practice B, 3 GPs and the PN participated. Each participating practitioner was asked to identify 2 or 3 patients meeting the study inclusion criteria and offer them the opportunity to participate in the study. The inclusion criteria were that patients should be aged between 30 and 65 years, and have at least two long-term conditions. The type of condition was not specified and could be mental or physical. Exclusion criteria were (a) unable to give informed consent including those with severe learning disability, severe active mental health problems (active psychosis, schizophrenia, bipolar illness, psychotic depression, severe depression including active suicidal ideation), severe dementia or other severe cognitive impairments, (b) terminally ill or considered by their GP as likely to die within next 12 months, and (c) unable to understand spoken and written English.

The focus of the pilot study was to explore patient selection, recruitment rates, and the delivery of the intervention in relation to system changes, training and delivery of support to practitioners, and the feasibility of data collection.

Following feedback from our work to develop the intervention, participating Practices were presented with the core ‘ingredients’ of the intervention but were allowed to adopt flexible approaches as to how they operationalised it. For example, it was left to the practice to decide who (GP or PNs) should deliver the longer consultations and provide continuity. Practitioners offering longer consultations were given a bespoke CARE Plus care plan in which to record the details of the consultations in relation to the pre-defined core ingredients and as an aid to providing the CARE Approach (available from corresponding author). They were also given a range of additional tools that they could use as they saw fit in navigating the consultation (available from corresponding author). Finally, they were also provided with copies of eight different self-management booklets, developed and published by Professor Chris Williams (Glasgow University), which they could give patients as they felt was appropriate. These booklets cover self-management of low mood, anger management, low motivation, alcohol problems, smoking cessation, weight loss, and coping with illness and disability, based on a cognitive-behavioural therapy (CBT) approach (see www.llttf.com).

Qualitative data on practitioners’ views and experiences were obtained throughout the pilot study in group discussions: 5 in Practice A and 2 in practice B. Data were also collected from the Care Plan. Discussions with practice staff were often done at routine practice meetings and were conversational in tone and at times included both facilitators (usually RO, on occasion SWM) as members of the group (more closely aligned to ‘action research’ than focus group interviews). This approach, therefore, was quite distinct from facilitation of a focus group interview, although it resulted in valuable data (field notes and interviews). These were mainly conducted in Practice A, as Practice B had problems with staff shortages during the pilot period and the GPs and PNs were often unavailable to attend meetings. With participating patients, qualitative data were obtained at the end of the study in six individual face-to-face interviews conducted by RO (three patients from each practice). These six patients had agreed to interview during the initial recruitment of patients to the pilot study. The qualitative data collection, management and analysis followed the same process as above.

Quantitative data (patient completed questionnaires) were collected from all participating patients but are not reported in this paper, which focuses on the qualitative data findings. However, we do report response rates to the baseline and follow-up questionnaire.

## Results

### Defining and developing the intervention

#### System changes: Time and relational continuity

The suggested system level changes involving longer consultations and enhanced relational continuity (seeing the same practitioner) was endorsed by all participants in the patient, patient representative and PN groups and by the majority of participants in the GP groups. The importance of relational continuity and a whole person-centred approach to care was also strongly endorsed by participants in all groups.

Views differed across and within groups as to who should provide the proposed longer consultations; GPs, PNs, Health Care Assistants, Support Workers or some combination of these practitioners were all suggested. There was also a range of views regarding how long the extended consultations needed to be. Several of the GPs suggested ‘double-appointments’ (20 min rather than the usual 10 min). PNs felt that substantially longer would be required to conduct a comprehensive holistic assessment. Patients and their representatives also felt that more than 20 min would be required to let them really ‘tell their story.’ In terms of how many extended consultations would be needed per patient, participants felt this would depend entirely on the individual case.

#### Practitioner training and support

Practitioner support was considered important in all groups. Practitioners had mixed views on how best to deliver this; some believed that training and support should be delivered at the individual practice level for all team members, whereas others highlighted the value of training across practices. The GPs and PNs found it hard to predict exactly *what* would be most helpful but identified the potential value of training that focused on how best to engage and motivate patients.

Both patient groups suggested that practitioners might benefit from training in listening skills.

Support in structuring the consultation was also recognised as important by many practitioners. The 5As model suggested in the original proposal was generally not popular; indeed the use of ‘toolkits’ generally were not favoured by the GPs who saw them as a ‘tick-box’ exercise. However, the CARE Approach (Connect–Assess–Respond–Empower) was, however, deemed to be a useful and simple model which could be used to guide the consultation and structure a care plan.

Whilst all groups identified the potential value of practitioner training to help patients self manage a range of problems including stress, the patient groups also expressed the view that practitioners themselves might benefit from stress management.

#### Self-management support

Changes at patient level, whereby self management was promoted through the use of simple materials and community resources, were generally considered important. However some scepticism about the ability of patients to use such material in the context of their social circumstances was expressed in the GP and PN groups. The idea of using mindfulness-based approaches to stress management with patients was popular among all participants, especially in the form of a CD for patients to listen to and practice at home. It was felt important to make any material simple and accessible to those with literacy problems.

### Overall

The findings of this study supported the view that the intervention should be comprehensive and take a whole-person approach. The essential structure of the practitioner/patient consultations to be utilised in such an approach were defined as comprising four key elements, which we framed within the CARE Approach:

1. Establishing and maintaining therapeutic relationships with patients (Connect),
2. Focusing on the ‘whole person’ in assessing health problems in terms of their individual personal and social contexts (Assess),
3. Responding in an empathic and validating way to problems (Respond), and
4. Empowering patients by helping them achieve realistic goals and improve self-management (Empower).

To support this, the key components of the intervention were defined as system changes to allow longer consultation time with relational continuity; training in the use of CARE Approach and support for practitioners, and self-management support for patients. On this basis we termed the intervention CARE Plus, which was then piloted in the two practices in the deprived areas.

### Piloting

The two practices identified 30 suitable patients for the pilot of the CARE Plus intervention, and 20 patients agreed to participate (14 from practice A and six from practice B). These comprised 12 females and 8 males, with a mean age of 50 years. From these 20, three (15%) did not attend any CARE Plus consultations, seven attended only one (35%) and ten attended two or more (50%). The mean number of CARE Plus consultations was 1.6 per patient. All patients who attended two or more CARE Plus consultations saw the same practitioner on each occasion.

#### Choosing the patients

There was similarity in patient selection by the different participating practitioners in terms of the mix of medical, social and psychological problems, although there were distinct reasons why each GP and PN had chosen particular individuals for CARE Plus (Box 1).

**Box 1.** Selection based on varying social and medical problems

*I’ve purposefully chosen them to be different eh eh in terms of what their backgrounds are and their problems and the demographics of them eh as well. They have all got … different problems and I did that purposefully because I didn’t want to do the same for everybody that that came in …it’s an experiment if you like.*	
(Practice B, Meeting 1, GP Participant 2)	
**Selection based on relationship continuity or perceived ‘readiness to engage’**	
*The first patient was basically an emergency patient who had a stroke recently …when I offered the slot it was a bit more about continuity of care. …(Second patient) is quite a strong character and again possibly I chose her because I felt she was ready to engage and change … (third) she is somebody we know parts of the family and none of them really engages a lot em and for me it’s more as well signalling ‘I’m your doctor now and you can come to me with anything.*	
(Practice A, Meeting 1, GP Participant 1)	
**Selection based on desire to find a better way of working with known patients**	
*They are all patients that I see regularly with em I feel I’ve got a long list of … deal of things I always want to deal with and never quite have time to deal with them all properly …But they are all they are all kamikaze (laughing) heart sink patients … It would be really nice to have more time to deal with them in a better way.*	
(Practice A, Meeting 1, GP Participant 3)	
**Invitations elicited mainly positive responses from patients**	
*Patients are very positive about it. I think I haven’t had single ones who has turned down the suggestion.*	
(Practice A, Meeting 1, GP Participant 1)	
*I have. But yeah …very near (laughing) and a few others. But that I think maybe the patients who have turned down have been intimated by the idea of research because of their own literacy problems … Or the fact that they just haven’t got, with the burden of illness, they haven’t got time to give us more time. They haven’t got time to spend more time with us or to spend time with a researcher.*	
(Practice A, Meeting 1, GP Participant 3)	
**Invitations to participate were repeated for some patients**	
*He was … adamant that he didn’t want to take part in this study but he still kept his appointment yesterday and basically the reason that he didn’t want to take part was he has literacy problems em and that’s a real barrier, and self-esteem issues as well.*	
(Practice A, Meeting 2, PN Participant 1)	

#### Practitioners’ experiences of the CARE Plus consultations

In relation to length of the consultations, practitioners described how it allowed them to explore patients’ backgrounds (e.g. family history), their current circumstances (e.g. relationships, housing, etc) as well as their medical problems (physical and psychological). Practitioners generally felt that the CARE Plus consultations had provided the opportunity to gain a new perspective on patients. Most were surprised at the length of time that the first CARE Plus consultation required (30–40 min). The value of having extra time, and how it was anticipated to be of benefit to patients, was frequently contrasted with the constraints practitioners experienced within their normal consultations. Recording the details of the intervention consultations in the care plan was also perceived to be time consuming and, consequently, the CARE Plus care plans were not consistently completed by all practitioners. Nevertheless, practitioners valued them as a ‘*record of progress*’ that were motivating to review at follow-up consultations.

#### Practitioner’ experience of the CARE Plus goal setting

The CARE Plus care plan guided practitioners to help the patients develop their own ‘plan of action’. Some of the approaches used to help patients identify or clarify their goals are presented in Box 2. Challenges with regard to setting realistic goals with multimorbid patients were common. Setting too many goals, and/or unattainable goals could demotivate patients. There was a perceived risk of using goal setting with some patients who were particularly sensitive to ‘pressure’. However, others felt that there was potential with some patients to use an ‘action plan’ to help them feel less overwhelmed and pressured to tackle everything at once:

He’s been struggling to kind of put together an idea of how he can actually achieve these kind of long term goals …  I find it quite useful … to basically try and use the long term goals as a target to kind of enable his ideas and then just break it up into much more shorter term ideas to kind of take it from there*.*

**Box 2.** Descriptions of goal setting

*From the people that I have seen as I say there’s nothing that I have done or the action plans that I’ve done which eh I have said ‘you need to this’ …I’d … felt … that the time spent with them I could then identify things that they know they need to do themselves and then just clarify it for them essentially. Just take … what’s out of half hours discussion and take it down to two or three points that they can just concentrate on themselves … They’ve also got a lot of long-term goals but they appreciate that they can’t be changed straight away. But small steps in the right direction.*	
(Practice A, Meeting 2, GP Participant 2)	
*I think that’s is where the extra time comes in that you can listen you are not stressed by going through your head ‘I need to do five issues …’. You can let them address their needs … You can listen connect and then bring (your) agenda in as well because I think at the end of the day I still feel quite …yeah, I am a doctor and I need to make sure that I get something through, which is important and not forgotten.*	
(Practice A, Meeting 2, GP Participant 1)	
*We worked out that (although I didn’t kind of plan it this way) eh it worked out over the course of the interviews that one would generally be something that they’ve already started to change themselves, one would be something that, that needs to eh that they know they need to change themselves, or have expressed desire to change during the course of the interview. And one was something that I kind of picked up on that might be beneficial for them which may both be such eh a success given that it’s a kind of an imposed goal if you like rather than something that they’ve picked up themselves. That’s kind of the way it turned out for me but without any particular eh eh notion of doing it that way in the first place.*	
(Practice A, Meeting 3, GP Participant 2)

Practice A, Meeting 3, GP Participant 2

#### Practitioners’ experience of continuity of care

One of the most critical aspects of the intervention, from the practitioners’ point of view, was the opportunity and importance of providing continuity to patients:

Without wanting to sound too arrogant we are quite often the only person that brings consistency and continuity and sometimes it’s this engaging, coming back, and being proud of something and we take over this paternal role in praising them …If you have a life where there is not a lot of positive items if you then can go to your doctor who says ‘you have done really well you should be proud of yourself’ it’s powerful. It gets lost if you don’t have any continuity*.*

Practice A, Meeting 2, GP Participant 1

Relationship continuity also had rewards for the GPs as they were able to closely follow-up, and receive feedback about improvements in self-management. One GP describes the feedback she got from one of her CARE Plus patients who had set herself a goal to give up smoking:

She said ‘this is the first time in sixty years I’ve done something for myself’ and to be quite honest I mean that has just mind blowing I find … that just puts it in a completely different dimension and shows how worthwhile it is what we are doing*.*

Practice A, Meeting 2, GP Participant 1

However, it was not uncommon for patients in this setting to have difficulties keeping appointments, with consequences for the practice in terms of time and appointment management. This seemed to raise more issues for the PNs than GPs who were able to find other tasks to do, such as administrative ones, during these gaps.

#### Practitioners’ experience on the CARE Plus peer support and training meeting

Due to time constraints, only one meeting was held during the pilot study, during which peer support was provided in sharing views and experiences in relation to the implementation of the CARE Plus intervention. During the meeting two specialist consultants in mental health attended, one delivering a talk and discussion on motivational interviewing, and the other on mindfulness-based approaches. Feedback from the meeting suggested that the practitioners valued the peer support and the mindfulness stress management technique, which they thought would be helpful for themselves as well as for the patients, but were less enthusiastic about motivational interviewing as a consultation technique.

#### Patients’ experiences of CARE Plus consultations

The CARE Plus consultations were generally very popular with patients. Most of the interviewed patients had no experience of being given time purely devoted to thinking about their problems and circumstances. One patient described how she usually felt rushed within consultations and, consequently, often forgot to raise some of her concerns, and how relaxed she felt knowing she was being given more time:

We were talking slowly. It didnae feel … See not feeling rushed that was the best …she would ask me something else, which would lead to me asking her something else … When I went in I took my jacket off because I knew I was there for thirty minutes … I got comfortable, I kinda just I knew I wasn’t in a rush and I knew she wasn’t typing out the prescription. So that’s it just made it feel more comfortable more relaxed.

Practice A, Patient Participant 4

Another patient who had a number of medical conditions explained how longer appointments had made it possible for him to talk about all his medical problems within one appointment, enabling his GP to ‘make connections’ never made before:

He knows my other problems but we’ve never really had time to discuss them up until that last visit [CARE Plus consultation] which was really good. I was in there I think for about fifty minutes … There were things that I’m having problems with and he’s saying ‘well look it could be this’… things that hadn’t even occurred to me (e.g. sleep apnoea) …When you go for an emergency appointment you can only talk to him about why you’re there. You cannae go into other detail. … That was a great session that I had with him you know. So I was really pleased about that*.*

Practice A, Patient Participant 1

Another benefit of having more time, that two patients raised, was being able to disclose problems they were ordinarily reluctant to talk about. One man explained that he had been worrying for a long time that he might have prostate cancer because of his urinary symptoms and despite his reluctance, he was able to raise this during his Care Plus appointment. Another patient felt the time he had spent with his GP had also made it easier for him to talk about his mental health issues.

#### Patients’ experiences of relational continuity in the CARE Plus consultations

Patients spontaneously raised the issue of continuity and how much they valued being able to see the same doctor each time they visited the practice for their CARE Plus consultations. All participants reported previous difficulties arranging appointments with a particular doctor of choice. By contrast, patients were able to plan their CARE Plus appointments in advance, ensuring they could return to see the same GP or PN again. The development of the relationship between doctor and patient, and what this meant in terms of supporting self management, was also highlighted within patient’s accounts. There were repeated references to the ‘mutual understanding’ developed within CARE Plus consultations, emphasising the time spent by the practitioner in getting to know them as people.

#### Patients’ experiences of the CARE Plus consultation goal setting

Some patients found goal setting, used during consultations to support self-management, particularly helpful. One patient described how she had felt ‘*stuck*’ and that setting a goal had been a ‘*push in the right direction*’ and had motivated her:

The anti-depressants definitely [helped]. But I think the fact that she's working with me …We are going to have a goal. She says ‘you need a goal. What's your goal?’ and I went ‘it’s my daughters 30^th^ next June … we will have a big party for her I says I want to get up and dance because I love dancing’. She went ‘right … that’s what our goal is going to be’… She's kind of going ‘ok, it will take a long time and it’s going to be a slow process, but we will get you there’. Well no-one has ever said that to me …She's kind of given me that push and interaction that I think I needed from somebody*.*

Practice B, Patient Participant 6

However, other patients had so much to deal with in their lives, along with medical problems, that they felt that trying to meet a particular goal was too difficult for them at the present time.

#### Patients experiences of self-help materials

Some of the patients interviewed had been given self-help booklets, written by Professor Chris Williams, that practitioners could use to compliment the CARE Plus Consultations. There were mixed views on the helpfulness of these:

I’ve read bits and pieces of it, you know … Sometimes they are a wee bit hard to believe … they are unrealistic … If you’ve got problems and you read though these books and you think ‘Jeez there’s shouldn’t be anything wrong with me at all’, you know …You’ve got to be realistic in the fact that disabilities does stop you from doing certain things …If you go from one page to the back page you get the feeling that ‘oh, I should be able to do all this stuff’ but you know you can’t. So I think you’ve just got to do it em small bits at a time*.*

Patient 1, Practice 1, GP 2

### Quantitative data collection

Patients were initially sent the questionnaire by post and then phoned on up to three occasions to encourage response. Of the 20 patients who were recruited into the pilot study, baseline questionnaire data was collected on 14 (70%). Follow-up questionnaires at 3 months followed the same regimen but were returned by only four out of the 14 patients (29%). Telephone discussions suggested that many of the patients found the questionnaire excessively long.

### Optimisation

The findings of the pilot study were used to further optimise the intervention, at all three levels (system; patient–practitioner interaction; self-management support). At system level, the length of time required in the first CARE Plus consultation was generally longer than many GPs envisaged, and averaged 30–40 min, with 20–30 min at follow-up consultations (although this varied according to the patient). The need for relational continuity was reinforced. The CARE Approach as the framework for the longer consultations was adopted and the associated CARE Plan was shortened (available from corresponding author). The training and support of practitioners was refined to combine peer support, personal and group goals for each training session, and a 30-min period of mindfulness-based stress reduction in each session. The self-management support material for patients was stream-lined to one self-help booklet written for people with long-term conditions, one short booklet about the mindfulness approach, and CDs explaining these and CDs providing guided mindfulness practices (spoken by a male and a female clinical psychologist). These changes are shown in Table 1, which draws on the TIDier checklist,^15^ and shows the final details of the CARE Plus Intervention. The questionnaire was also substantially shortened and a strategy developed by the programme manager (BF) to ensure higher levels of baseline and follow-up data collection in the future.

**Table 1.** Details of the final iteration CARE Plus intervention.

Intervention aspects		Details:	
		In this paper	Other/comments
Intervention name	CARE Plus: a primary-based whole system complex intervention (CARE Plus) for patients with multimorbidity living in areas of high socioeconomic deprivation.	This paper describes the development and optimisation of the intervention and the final iteration to be tested in a phase 2 exploratory RCT.	This work is part of a 4-year programme of research called ‘Living Well with Multimorbidity’ funded by the Chief Scientis Office of the Scottish Government NHS Applied Research Programme Award (ARPG07/01).
WHY (theory, background, essential elements)	Likely key components drawn from literature, expert opinion, and views of patients, representatives and health care staff.	This papers outlines the background to the research and how the key components of the CARE Plus intervention were identified and developed.	See Bamett et al.^2^ and O’Brien et al.,^7,8^ for details of the baseline epidemiology and qualitative research that informed the intervention development.
WHAT (materials)	Longer consultations using bespoke CARE Plus care plan; practitioners training and support materials; patient self-management support materials.	The key components of the intervention are presented in the paper and the final iteration is outlined under the ‘optimisation section.’ In the pilot, a variety of CBT-based booklets were given to different patients. Mindfulness-CDs were not available at the time of the pilot.	A final version of the CARE Plan will be developed for the RCT. The final version of the materials given to practitioners in the training days and to the patients in the RCT will be developed before the RCT begins, informed by the current findings of this paper. The patient self-management support pack requires further development before the RCT.
WHO will provide intervention	CARE Plus consultations Practitioner training and support	This paper identified the need for flexibility in terms of who delivers the intervention (GP or PN) and that different practices will be allowed to operationalise this differently.	The practitioner training and support in the pilot study was delivered by an academic GP (SWM) and a psychiatrist skilled in CBT and mindfulness. This will also be the case in the exploratory trial.
HOW (modes of delivery)	Patient consultations will be face-to-face with GP or PN Practitioner training will be group based.	In the pilot study the CARE Plus consultations were all held face-to-face.	In the exploratory trial, practitioners will deliver the consultations face-to-face for reasons of efficiency.
WHERE (locations)	Patients consultations Practitioner training and support.	In the pilot study almost all the consultations were delivered in the practices. Practitioner training and support was only delivered in one practice, and this took place in the practice.	In the exploratory trial consultations will take place in the practices. Group training and support will involve all GPs and PNs in the intervention group meeting 3–4 times over 12 months in a single location.
WHEN and how much	Number of CARE Plus consultations per patients. Practitioner training and support meetings: maximum 4 meetings; 3 h per meeting. Patient self-management support material.	In the current paper, it was clear that the length of the consultations required was contested in the development phase; however, in the pilot in the 2 practices, it was agreed that the initial consultation needs 30-40 min and the follow-up 20-30 min. The number of follow-up consultations required was not established. In this paper, only 1 practice took part in the training and support and had only 1 meeting of 3 h.	In the exploratory trial we will recommend that practitioners see the selected patients in the CARE Plus intervention at least twice, and that the initial consultation will require 30–40 min and follow-up 20–30 min. Further follow-up consultations will be at GP/PNs discretion depending on patients’ needs and progress, as in the pilot. Practitioner training and support meetings: In the RCT we will aim for 3–4 meetings, 3 h per meeting, over the 12 months of the trial. Patient self-management support pack will be given to patients by the practitioners and used at the patients’ own discretion.
Tailoring	GPs/PNs allowed flexibility to tailor to patients’ needs but core components essential.	As above, the core ingredients as described in the paper are fixed but discretion is allowed as to who delivers, and how often.	As above
Modifications	To CARE Plus consultations, practitioner training and support and/or patient self-management pack.	Several modifications were made during the development and optimization as outlined in this paper	Further modifications required before exploratory RCT as above.

## Discussion

### Main findings

The current work was part of our programme of research called ‘Living Well with Multimorbidity,’ and was the second iteration of the development of a whole system primary care-based complex intervention for multimorbid patients living in the deprived areas. It adds to, and builds on, our earlier work-stream of the programme in which we defined the target population,^2^ and gathered important ‘baseline’ views and suggestions of practitioners and patients working and living in the deprived areas in terms of living well with multimorbidity and how primary care might better respond.^7,8^

We have further developed and optimised the intervention in the current study, which we have named CARE Plus, which aims to enhance quality of life in multimorbid patients living in the deprived areas. CARE Plus involves system change (longer consultations with relational continuity), patient–practitioner interaction change (an empathic patient-centred structured approach), training and support for staff to deliver this and support for patient self-management. We have also revised the patient questionnaire and standard operating procedures to try to ensure higher response rates in the future. The intervention is now ready to be evaluated in a phase 2 cluster-randomised trial to establish proof of concept, establish broader feasibility, and estimate intervention impact to inform power calculations for a phase 3 cluster-randomised trial.

### Strengths and weaknesses

An important feature of the intervention development was its ‘co-design’ with practitioners, patients, and patients representatives. The fact that this co-design was planned from the very start was, on reflection, a very important factor in the programme, and the duration of the programme meant that meaningful relationships and discussion could be held with all the key stakeholders. Similar to our earlier findings,^7,8^ participants readily recognised the complex problems associated with living with multimorbidity in the deprived areas and the challenges these raised for patients and their primary care practitioners. Patients and their representatives fully endorsed the need for longer consultations and relational continuity of care. They also recognised the pressures on health care staff and supported the case for support and training of primary care staff. The GPs and PNs also saw the need for targeted longer consultations and a holistic approach to the care of multimorbid patients, again in line with our previous findings^7^ and agreed that training and support was required. Most participants also welcomed the idea of additional self-management support, though stressed the need to make such material accessible and relevant to the needs of multimorbid patients in the context of deprivation, and poor literacy.

The pilot study showed that the intervention could be implemented in practice in terms of the practices identifying eligible patients and providing them with longer consultations and using the care plan, and trying out a variety of patient self-help materials. However, due to delayed time-lines in the programme, we were only able to run the pilots for 3 months (we had originally envisaged much longer than this) and thus were not able to assess patient qualitative outcomes longitudinally. Perhaps due to this shortened time-frame, not all patients received a second CARE Plus consultation, although relational continuity was achieved for those who did. In addition, only one practice (Practice A) had a training and support session for the practitioners even though we had originally envisaged having more than this. Practice B, due to staff shortages within the practice at the time were not able to fully participate in the pilot, as it proved very difficult to arrange meetings between the GPs and the researchers. It was also not possible to arrange any training and support sessions. However, generally the intervention was well received in both practices. The pilot work was also helpful as it also highlighted the need for flexibility and led to several relatively small but important changes including a revised CARE Plan, the inclusion of mindfulness-based stress reduction techniques in the support of practitioners and patients, and the stream-lining of the written self-management support (SMS) material for patients. It was also an important opportunity to test and modify the patient CARE Plan and the patient questionnaire, and to devise a strategy to ensure higher response rates to patient questionnaires in the future phase 2 trial.

### Relationship with published literature

The rationale for the components of this whole-system approach was supported by direct or indirect evidence as far as possible, as well as the views of the participants. The increased prevalence and burden of multimorbidity in the deprived populations need to be considered in the context of the ‘inverse care law,’ which states that the availability of good medical care tends to vary inversely with the need for it in the population served.^16,17^ Primary care has a central role in the management of multimorbidity, but the continuing existence of the ‘inverse care law’ limits this potential in the deprived areas due to the mismatch between patients needs and primary care capacity.^18,19^ Consultations in the deprived areas are shorter than in more affluent areas^18,20^ yet patients have more complex problems to discuss due to more mental, physical and social problems.^18^ The GPs working in the deprived areas suffer more burn-out^21^ and feel more stressed in the consultations.^18^ Patients with complex problems are less enabled by these consultations compared with their counterparts in more affluent areas^18^ and have worse outcomes.^22^

In terms of the benefit of longer consultations, the international evidence-base is limited^23,24^ but in the context of high deprivation areas in Scotland we have previously found in a single practice that extended consultation length was associated with more enablement for complex patients and decreased GP stress.^25^ Relational continuity is important to patients with complex needs in the deprived areas.^26^ Empathic patient-centred care predicts patient enablement and better health outcomes^27–29^ but the GPs tend to be less patient-centred with patients of lower socioeconomic status.^22,30^ Finally, patients’ self-management support for managing the stress of living with long-term conditions can be helpful in improving outcomes.^31^

There are few complex intervention which have been specifically developed for patients with multimorbidity, especially in the context of socioeconomic deprivation.^9^ A recent large primary care-based RCT that aimed to enhance self-management in general practice in a relatively high deprivation setting with multimorbid patients failed to show any benefit.^32^ However, it did not include longer consultation time with the GPs. The CARE Plus intervention, if effective, may be cost-effective (if it improves quality of life above usual care) as it does not involve the employment of new staff or therapists but builds on the generalist skills of existing primary care staff.^33^

### Implications

In line with guidelines on the development of complex interventions,^10^ we have developed and optimised a whole system primary care-based intervention (CARE Plus) to enhance quality of life for multimorbid patients living in the very deprived areas. The likely effectiveness, cost effectiveness and feasibility of this approach is ready to be tested in an exploratory cluster randomised controlled trial.

## References

[1] Violan C Foguet-Boreu Q Flores-Mateo G Prevalence, determinants and patterns of multimorbidity in primary care: a systematic review of observational studies. PloS one 2014; 9: e102149–e102149. 25048354 10.1371/journal.pone.0102149 PMC4105594

[2] Barnett K Mercer SW Norbury M Epidemiology of multimorbidity and implications for health care, research, and medical education: A cross-sectional study. Lancet 2012; 380: 37–43. 22579043 10.1016/S0140-6736(12)60240-2

[3] Salisbury C Johnson C Purdy S Epidemiology and impact of multimorbidity in primary care: A retrospective cohort study. Br J Gen Pract 2011; 582: e12–21. 10.3399/bjgp11X548929 PMC3020068 21401985

[4] Lawson K, Mercer S, Wyke S, et al. Double trouble: The impact of multimorbidity and deprivation on preference-weighted health related quality of life - a cross sectional analysis of the Scottish Health Survey. *Int J Equity Health* 2013; 12. 10.1186/1475-9276-12-67 PMC3765174 23962150

[5] McLean G Gunn J Wyke S The influence of socioeconomic deprivation on multimorbidity at different ages. Br J Gen Pract 2014; 64: e440–447. 24982497 10.3399/bjgp14X680545 PMC4073730

[6] Payne R, Abel G, Guthrie B, et al. The impact of physical multimorbidity, mental health conditions and socioeconomic deprivation on unplanned admissions to hospital: A retrospective cohort study. *CMAJ* 2013; 185: E221–228. 10.1503/cmaj.121349 PMC3602270 23422444

[7] O’Brien R Wyke S Guthrie B An “endless struggle”: A qualitative study of GPs’ and Practice Nurses’ experiences of managing multimorbidity in socio-economically deprived areas of Scotland. Chronic Illn 2011; 7: 45–59. 20974642 10.1177/1742395310382461

[8] O’Brien R Wyke S Watt G The ‘everyday work’ of living with multimorbidity in socio-economically deprived areas of Scotland. JOC 2014; 9: 62–62. 10.15256/joc.2014.4.32 PMC5556407 29090148

[9] Smith SM Soubhi H Fortin M Interventions for improving outcomes in patients with multimorbidity in primary care and community settings: Systematic review. BMJ 2012; 345: e5205–e5205. 22945950 10.1136/bmj.e5205 PMC3432635

[10] Craig P Dieppe P Macintyre S Developing and evaluating complex interventions: The new Medical Research Council guidance. BMJ 2008; 337: a165–a165. 10.1136/bmj.a1655 PMC2769032 18824488

[11] Glasgow RE Emont S Miller DC Assessing delivery of the five ‘As’ for patient-centered counseling. Health Promot Int 2006; 21: 245–255. 16751630 10.1093/heapro/dal017

[12] Fitzgerald N Heywood S Bikker AP Enhancing empathy in healthcare: Mixed-methods evaluation of a pilot project implementing the CARE Approach in primary and community care settings in Scotland. J Compassionate Healthcare 2014; 1: 6–6.

[13] Bikker AP Cotton P Mercer SW Embracing empathy in healthcare. A universal approach to person-centred, empathic healthcare encounters, London-New York: Radcliffe, 2014. 23232141

[14] Ritchie J Lewis J Qualitative research practice, London: Sage, 2003.

[15] Hoffmann TC Glasziou PP Boutron I Better reporting of interventions: Template for intervention description and replication (TIDieR) checklist and guide. BMJ 2014; 348: g1687–g1687. 24609605 10.1136/bmj.g1687

[16] Hart JT The inverse care law. Lancet 1971; 297: 405–412. 10.1016/s0140-6736(71)92410-x 4100731

[17] Watt G The inverse care law today. Lancet 2002; 360: 252–254. 12133675 10.1016/S0140-6736(02)09466-7

[18] Mercer S Watt G The inverse care law: Clinical primary care encounters in deprived and affluent areas of Scotland. Ann Fam Med 2007; 5: 503–510. 18025487 10.1370/afm.778 PMC2094031

[19] Mercer SW Guthrie B Furler J Multimorbidity and the inverse care law in primary care. BMJ 2012; 344: e4152–e4152. 22718915 10.1136/bmj.e4152

[20] Furler JS Harris E Chondros P The inverse care law revisited: Impact of disadvantaged location on accessing longer GP consultation times. Med J Aust 2002; 177: 80–83. 12098344 10.5694/j.1326-5377.2002.tb04673.x

[21] Pederson AF Vedsted P Understanding the inverse care law: A register and survey-based study of patient deprivation and burn-out in general practice. Int J Equity Health 2014; 13: 121–121. 25495229 10.1186/s12939-014-0121-3 PMC4272764

[22] Jani B Bikker AP Higgins M Patient-centredness and the outcome of general practice consultations with patients with depression in areas of high and low deprivation. Br J Gen Pract 2012; 62): e576–e581. 22867682 10.3399/bjgp12X653633 PMC3404336

[23] Wilson AD Childs S Effects of interventions aimed at changing the length of primary care physicians’ consultation. Cochrane Database Syst Rev 2006; (1): CD003540–CD003540. 10.1002/14651858.CD003540.pub2 16437458

[24] Hutton C Gunn J Do longer consultations improve the management of psychological problems in general practice? A systematic literature review. BMC Health Serv Res 2007; 7: 71–71. 17506904 10.1186/1472-6963-7-71 PMC1890290

[25] Mercer SW Fitzpatrick B Gourlay G More time for complex consultations in a high deprivation practice is associated with increased patient enablement. Br J Gen Pract 2007; 57: 960–966. 18252071 10.3399/096016407782604910 PMC2084135

[26] Mercer SW Cawston PG Bikker AP Patients’ views on consultation quality in primary care in an area of high deprivation: A qualitative study. BMC Fam Med 2007; 8: 22–22. 10.1186/1471-2296-8-22 PMC1857696 17442123

[27] Mercer SW Neumann M Wirtz W Effect of General Practitioner empathy on patient enablement, and patient-reported outcomes in primary care in an area of high socio-economic deprivation in Scotland - A pilot prospective study using structural equation modelling. Patient Educ Couns 2008; 73: 240–245. 18752916 10.1016/j.pec.2008.07.022

[28] Little P Everitt H Williamson I Observational study of effect of patient centredness and positive approach on outcomes of general practice consultations. BMJ 2001; 323: 908–911. 11668137 10.1136/bmj.323.7318.908 PMC58543

[29] Derksen F Bensing J Lagro-Janssen A Effectiveness of empathy in general practice: A systematic review. Br J Gen Pract 2013; 63: e76–84. 23336477 10.3399/bjgp13X660814 PMC3529296

[30] Verlinde E De Laener N De Maesschalck S The social gradient in doctor-patient communication. Int J Equity Health 2012; 11: 12–12. 22409902 10.1186/1475-9276-11-12 PMC3317830

[31] Taylor SJC, Pinnock H, Epiphaniou E, et al. A rapid synthesis of the evidence on interventions supporting self-management for people with long-term conditions: PRISMS – Practical systematic Review of Self-Management Support for long-term conditions. Southampton (UK): NIHR Journals Library, December 2014. 25642548

[32] Kennedy A Bower P Reeves D Implementation of self management support for long term conditions in routine primary care settings: A cluster randomised controlled trial. BMJ 2013; 346: f2882–f2882. 23670660 10.1136/bmj.f2882 PMC3652644

[33] Reeve J, Dowrick CF, Freeman GK, et al. Examining the practice of generalist expertise: A qualitative study identifying constraints and solutions. *JRSM Short Rep* 2013; 4. 10.1177/2042533313510155 PMC3899736 24475347

